# Nanoplastic concentration and potential transport in the Arctic Ocean

**DOI:** 10.1038/s44454-025-00024-y

**Published:** 2026-01-09

**Authors:** Huiwen Cai, Charlotte Carrier-Belleau, Caroline Guilmette, Philippe Massicotte, Adèle Luthi-Maire, Julien Gigault

**Affiliations:** Takuvik Laboratory, IRL3376 CNRS-Université Laval, Quebéc, G1V 0A6 QC Canada

**Keywords:** Ecology, Ecology, Environmental sciences, Ocean sciences

## Abstract

Plastic contamination in the Arctic originates from local anthropogenic activities and long-range transport mediated by oceanic and atmospheric circulation, with nanoplastics representing a major emerging concern. Yet, quantitative data on their environmental distribution remain scarce. Here, we present the first multi-matrix, multi-site, and multi-year assessment of nanoplastics distribution in the Arctic Ocean. We found that polystyrene, polypropylene, and polyethylene nanoplastics are widely distributed across different matrices from the Svalbard region to the central Arctic Ocean. The total concentrations of polystyrene and polypropylene ranging from 10 up to 900 ng L⁻¹. Coastal areas may be more influenced by local human activities, whereas remote regions are likely affected by atmospheric transport and inputs associated with the Transpolar Drift. The extent to which each source contributes remains uncertain and may vary spatially. Differences in distribution across environmental matrices were also observed, with higher concentrations in snow and surface ice than at the ice-sea interface. The sea ice act as a temporary reservoir and secondary source of nanoplastics through redistribution processes across the snow-ice-seawater interface. These findings indicate the necessity for multi-scale, multi-temporal, and multi-spatial investigations, which are crucial for understanding the sources, transport pathways, and environmental fate of nanoplastics within the Arctic ecosystem.

## Introduction

Plastic pollution is a global issue, with 19–23 million tons of plastic waste entering aquatic ecosystems each year^1^. This contamination changes key biogeochemical processes that regulate climate resilience. The production, mismanagement, and degradation of plastics release greenhouse gases, while small plastic particles could influence the carbon pump, nutrient cycling, and deoxygenation trend^2,3^. These processes collectively weaken the ability of ecosystems to sequester carbon and adapt to ongoing climate change^4^. Plastics have the potential to degrade into smaller particles, forming microplastics (1 µm–5 mm) and nanoplastics (<1 µm)^5^.

Although polar regions have long been regarded as pristine and largely unaffected by direct human activities, various pollutants are now widespread, even in areas with no apparent human presence^6,7^. Microplastics have been detected in Arctic sea water^6,8^, sediment^9,10^, sea ice^11,12^, and snow^13^. Microplastics tend to fragment to nanoplastics, which potentially have much higher particle concentration. The abundance of microplastics at 11 µm dominates over 65% of a whole size fraction of microplastics in the Arctic^8^, which indicates the higher importance of studying small plastic particles. Owing to their smaller size and higher surface reactivity, nanoplastics can cross biological membranes, accumulate within cells and organelles, and induce oxidative stress and molecular damage, thereby posing greater risks to wildlife than microplastics^14,15^. The influx of nanoplastics may also increase environmental stress in the Arctic, which is already under severe pressure from rising sea temperatures and ocean acidification^16^.

Currently, very limited data exist on the occurrence and concentrations of nanoplastics in the environment, particularly in the Arctic Ocean. This is primarily due to the remoteness of these regions and the inherent challenges in routinely sampling and analyzing nanoplastics^17^. Among the various techniques to characterize trace analysis of nanoplastics^18^, pyrolysis gas chromatography-mass spectrometry (pyr-GC-MS) is one of the most robust and sensitive techniques which has been applied to detect nanoplastics in environmental samples, such as seawater from the North Atlantic subtropical areas^19^, sand from Caribbean Island shorelines^20^, and agricultural soils from central France^21^. Pyr-GC-MS was selected for this study to detect three representative plastics, including polystyrene, polypropylene, and polyethylene, which are among the most widely produced plastics globally. Based on their densities (polystyrene ≈1.05 g cm⁻³, polypropylene ≈0.91 g cm⁻³, and polyethylene ≈0.94 g cm⁻³), these plastics are likely to occur predominantly within surface ocean environments.

In this study, we sampled, extracted and analyzed nanoplastics in seawater, ice cores, melt pond water, and snow across the Arctic Ocean. We collected samples in July and August of 2022 and 2023 in an area bordered by Svalbard archipelago to the North Pole. This study discusses (i) the occurrence and concentration of nanoplastics from different matrices and (ii) potential sources and transport behaviors of nanoplastics in the Arctic Ocean.

## Results and discussion

### Nanoplastics identification and quantification in the Arctic Ocean sea surface

**Fig. 1:**
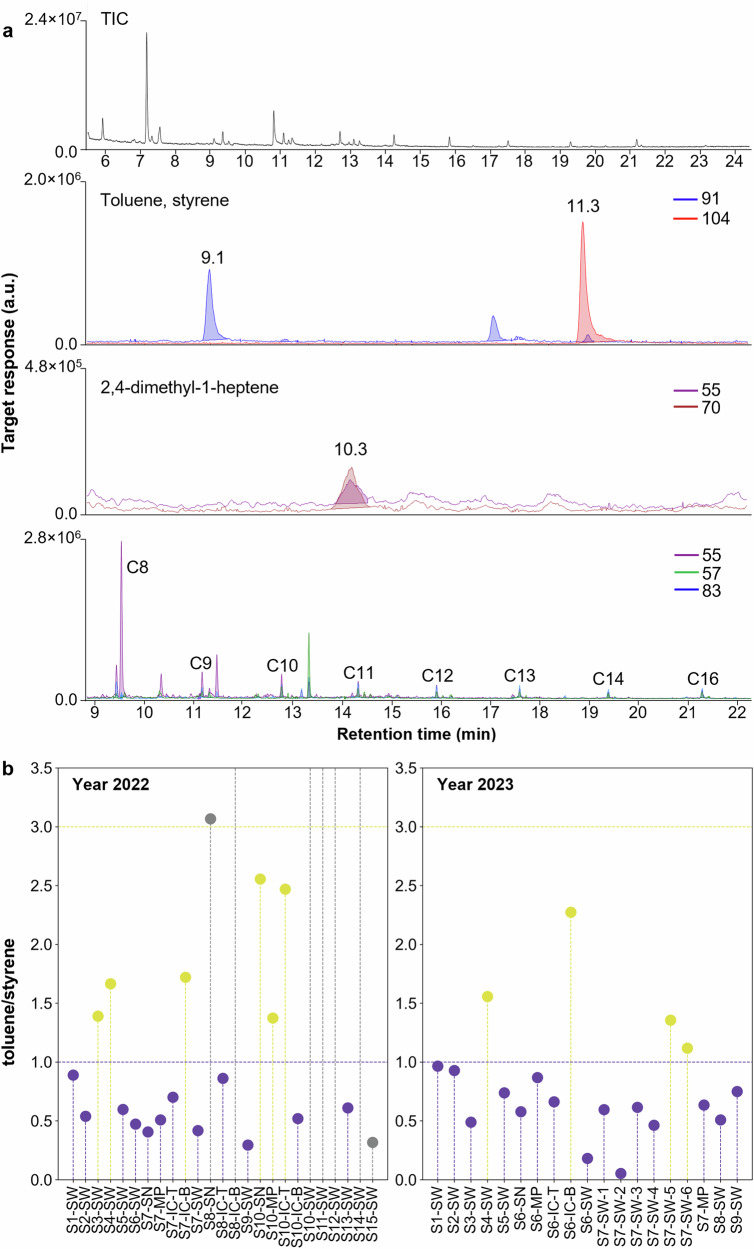
Identification of different nanoplastics in samples. **a** Typical total ion chromatogram (TIC) and extracted ion chromatograms (EIC) of selected indicator ions for polystyrene (*m/z* 91, toluene; *m/z* 104, styrene), polypropylene (*m/z* 55 and 70, 2,4-dimethyl-1-heptene), and polyethylene (*m/z* 55, 57, 83, n-alkadiene/n-alkene/n-alkane) nanoplastics; **b** toluene/styrene ratios in samples from 2022 and 2023. SW seawater, IC-T top of ice core, IC-B bottom of ice core, MP melt pond, SN snow.

Polystyrene, polypropylene, and polyethylene nanoplastics were detected at all 24 sampling sites. Nanoscale polymer identification and quantification were achieved by selecting characteristic pyrolysis products (i.e., pyrolyzates) after pyr-GC-MS detection, based on established studies^17,22^. These characteristic pyrolyzates included toluene, styrene, 2,4-dimethyl-1-heptene, and triplets of n-alkadiene, n-alkene, and n-alkane with a bimodal distribution, which served as markers for main polymers (polystyrene, polypropylene, and polyethylene) (Fig. 1a).

A major challenge in nanoplastic analysis is the interference from natural organic matter (NOM), which can lead to inaccurate polymer identification. To mitigate this, we applied the toluene/styrene ratio as an effective threshold to differentiate NOM contributions from polystyrene^22^. Previous data suggest that a ratio less than one is considered to be contribution by polystyrene, and a ratio greater than three is attributed to a large contribution by NOM^22^. Toluene/styrene ratios below 1 indicate the presence of polystyrene in most samples (Fig. 1b).

Polypropylene was identified by first confirming the retention time with the polypropylene standard, followed by applying an ion ratio of m/z 70/55 > 1, based on the NIST MS library pyrogram of 2,4-dimethyl-1-heptene to verify the polymer and exclude potential non-polypropylene interferences. The ratio was further verified using a series of mixed polystyrene and polypropylene standards (Table S3). For polyethylene, we observed the n-alkene series up to C14 with the triplet of n-alkadiene, n-alkene, and n-alkane, indicating the presence of polyethylene (Fig. 1a). With the polymer types verified, we determined the mass concentration through internal calibration procedures that we developed (Fig. S4 and Table S4). In this study, total nanoplastic concentration represents the sum of polystyrene and polypropylene nanoplastics. Quantitative analysis of polyethylene was not performed, as the method for polyethylene quantification at the nano scale is still under development. However, the qualitative identification demonstrates the occurrence of polyethylene pollution in the Arctic Ocean and calls the urgent need for further methodological advancement and field research on nanoplastic contamination in polar environments.

### Occurrence and distribution of nanoplastics in the Arctic Ocean

**Fig. 2:**
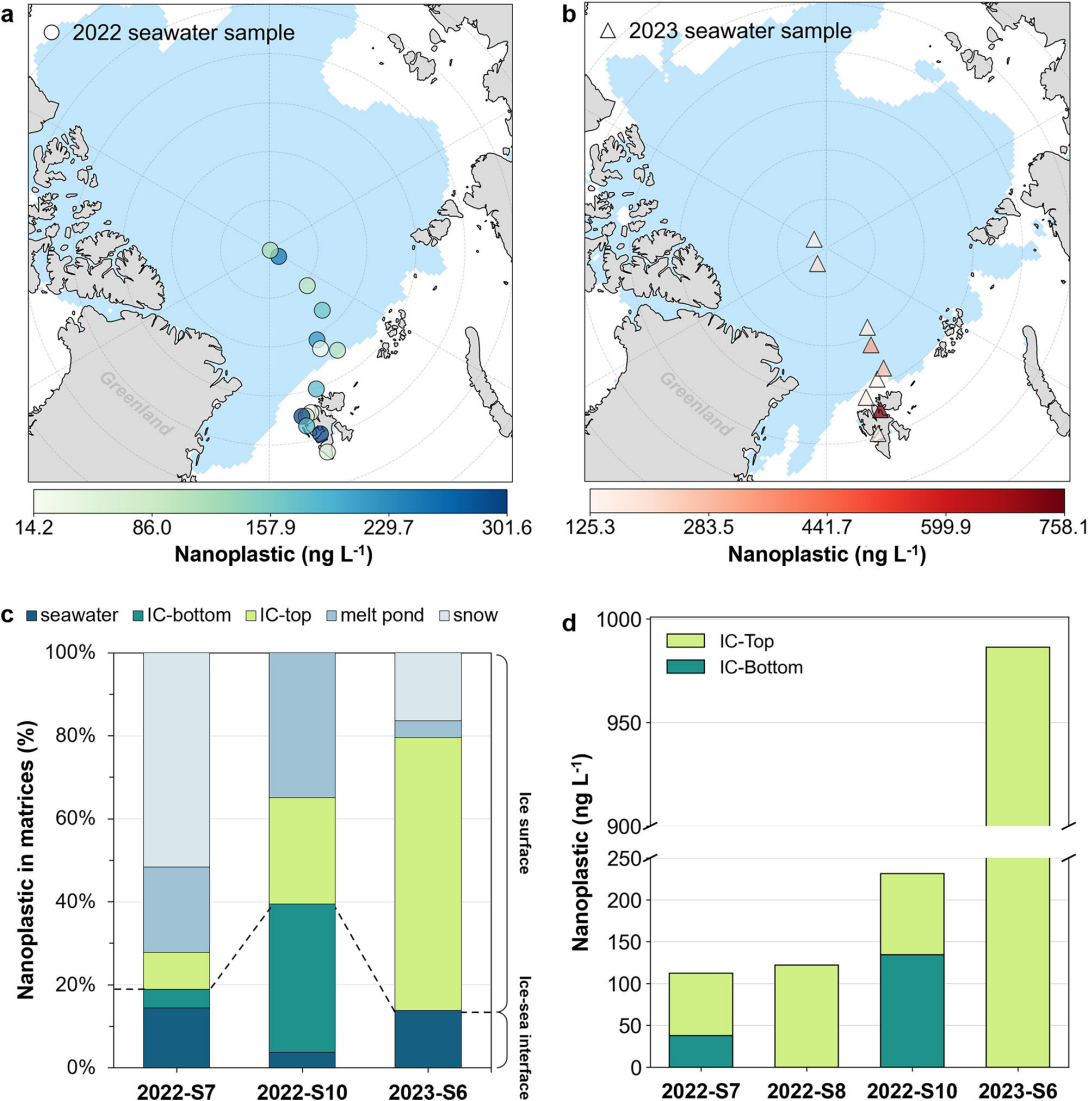
Distribution of polystyrene and polypropylene nanoplastics in the Arctic Ocean. The color blue represents the monthly sea ice concentration in August for 2022 (**a**) and 2023 (**b**). Circles represent sampling sites of seawater in 2022 (**a**), and triangles represent sampling sites of seawater in 2023 (**b**). The color gradient of the sites indicates the total concentration of polystyrene and polypropylene nanoplastics at each site. **c** Shows the proportions of nanoplastic concentrations among four environmental matrices, including seawater, melt pond, snow, and ice core (IC), collected during each expedition. **d** Presents the concentrations of nanoplastics in four ice cores, divided into top and bottom sections. No seawater sample was collected at site 2022-S8. Sea ice data were obtained from the National Snow and Ice Data Center^29^.

To date, there is no data on nanoplastic concentrations across the Arctic Ocean as we obtained. To ensure the reliability of our measured concentrations, we compared them with published microplastic data. Microplastic concentrations reported in surface seawater (0–20 m)^23^, with particle sizes around 0.1–0.25 mm, range from 1 to 30 particles m^-3^. Assuming an average plastic density of 1.05 g cm^-3^ and a spherical particle shape, this translates to a concentration range of about 0.5–250 ng L⁻¹. Considering the abundance difference among size fractions, particles smaller than 11 µm (excluding the nanometric fraction) are 65 times more abundant than those in the 0.1–0.25 mm fraction, indicating a higher contribution of nanoplastics in the Arctic^8^. Using these data, we estimate a concentration range from roughly 30 ng L⁻¹ to 16 µg L^-1^. Our findings of nanoplastic concentrations in matrices (~10–900 ng L⁻¹) align with this range, suggesting the potential predominance of nanoplastics in the Arctic Ocean (Fig. 2).

Nanoplastics were detected in surface seawater, ice cores (top and bottom parts), melt pond water, and snow during the 2022 and 2023 expeditions. Their concentrations in seawater showed clear spatial and interannual variability (Fig. 2a, b), generally decreasing with increasing distance from the Svalbard archipelago. Concentrations ranged from 14.2 to 301.6 ng L⁻¹ in 2022 (mean 150.0 ng L⁻¹) and from 125.3 to 758.1 ng L⁻¹ in 2023 (255.1 ng L⁻¹). The average nanoplastic concentration across all matrices increased from 159.8 ng L⁻¹ in 2022 to 345.4 ng L⁻¹ in 2023 (Table S4). Because most residents on Svalbard archipelago reside along the western shoreline, we categorized sampling sites into two groups: coastline sites and ocean sites, to evaluate nanoplastic concentrations influenced by different sources. Coastline sites are located closer to human activities, including that south of the site 2023-S3 (Fig. S1), where anthropogenic impacts are more direct. In contrast, ocean sites are farther from residential areas, primarily north of site 2023-S3 (Fig. S1), and are more influenced by long-range transport of nanoplastics.

At the coastline sites, local human activities likely contribute to the spatial heterogeneity of nanoplastic concentrations. Svalbard archipelago supports concentrated human presence and maritime traffic, particularly in Longyearbyen and Ny-Ålesund^24^. The archipelago experiences high seasonal intensity of maritime traffic associated with fishing, cargo transport, scientific expeditions, and cruise tourism every year^25^. Despite Longyearbyen being both a population and shipping hub, did not exhibit the highest nanoplastic concentrations. In 2022, the maximum concentration (301.6 ng L⁻¹) occurred at S13, located along the coast of Northwest Spitsbergen National Park, slightly higher than S1 (Longyearbyen Port, 297.1 ng L⁻¹). In 2023, the highest concentration was found at S9 near Alkefjellet (758.1 ng L⁻¹), whereas S1 (Longyearbyen) exhibited a markedly lower value (125.3 ng L⁻¹). Spatially, sampling sites at where residential density is higher, generally exhibited increased nanoplastic levels compared with the southern and northern coasts, which are sparsely inhabited. It is consistent with onshore observations on plastic debris at some sites, where lower plastic debris counts on southern beaches and higher along the Hinlopen Strait, suggesting that similar anthropogenic pressures on both terrestrial and marine environments^26^. In contrast, the interannual differences in nanoplastic concentrations cannot be explained solely by changes in population size or ship traffic volume^24,25^. Other dynamic factors, such as oceanic circulation, riverine input, and atmospheric deposition, may contribute to the instantaneous concentration differences observed among sites. To better understand these influences, multi-year and process-oriented investigations integrating oceanographic circulation, atmospheric deposition, and riverine input data are required to capture the temporary variability and mechanisms driving nanoplastic distribution around the archipelago.

Ship tracks further indicate that only a limited number of voyages extended northward from Svalbard toward the central Arctic Ocean^7^, making direct ship-related inputs an unlikely dominant source of nanoplastics in the open ocean. Consistent with this, the average concentration of nanoplastics decreased with increasing distance from Svalbard in both years (Table S5), which is compatible with stronger contributions in coastal and archipelago-proximal environments relative to the central basin. Atmospheric transport likely plays a more prominent role than ocean circulation in shaping nanoplastic distributions in the open Arctic Ocean. This hypothesis is supported by previous findings that atmospheric pathways deliver anthropogenic particles such as black carbon and microplastics to Arctic ecosystems^27,28^. In addition to atmospheric deposition input, sea ice may also act as an important role to instantaneous distribution of nanoplastics in the open Arctic Ocean. Interannually, the average concentration at ocean sites in 2023 was ~70 ng L^-1^ higher than in 2022. In August 2023, sea ice extended further south toward the Svalbard region than in 2022^29^, and sea ice concentration at the sampling sites increased by 6.8% (Table S7). The porous structure of sea ice allows the exchange of organic matter between seawater and atmosphere, while also enabling vertical transport of nanoplastics through processes such as air-ice gas exchange and brine drainage^30^. As reported in a previous study, most nanoplastics are expelled with brine during ice formation, the rest are trapped in brine pockets^31^. Consequently, enhanced ice formation may increase the release of nanoplastics into seawater, as newly forming ice can concentrate particles previously deposited from the atmosphere before subsequently releasing them during brine rejection^28,32^. Sea ice likely functions as a transient reservoir that both stores and releases nanoplastics, acting as a secondary source and a temporary sink simultaneously in the Arctic surface ocean.

Moreover, the contrast in the vertical profile signals, where nanoplastic levels are higher at the snow-ice interface than at the ice-sea interface (Fig. 2c), indicates a substantial contribution from atmospheric deposition. Vertical sections of matrices, from air deposition to seawater, further illustrate the instantaneous distribution of nanoplastics at three specific ocean sites: 2022-S7, 2022-S10, and 2023-S6 (Fig. 2c). At site 2022-S7, 51.6% (432.3 ng L^-1^) of the nanoplastics were detected in the snow and 20.6% (172.6 ng L^-1^) in the melt pond. At site 2022-S10, nanoplastics nanoplastics were mainly retained in the ice core (231.4 ng L^-1^) and melt pond (131.2 ng L^-1^), with only 14.2 ng L^-1^ detected in the seawater. At site 2023-S6, most nanoplastics were concentrated in the top part of the ice core (986.3 ng L^-1^), accompanied by a decreased level in snow (245.1 ng L^-1^). A similar vertical distribution was observed at site 2022-S8, where nanoplastics were detected only in the top part of ice core (Fig. 2d). In this study, nanoplastic concentrations were higher in surface compartments, such as snow, melt ponds, and the top of the ice core, than in sub-surface compartments, including the bottom of the ice core and surface seawater. This difference suggests a partial shift in contribution of nanoplastic sources and pathways beyond land and sea currents, with snow potentially acting as a transport or deposition source to sub-surfaces. The magnitudes of nanoplastic concentrations observed here, reaching up to 986.3 ng L^-1^ in surface ice and 432.3 ng L^-1^ in snow, are comparable to previously reported values of ~680 ng L⁻¹ for polystyrene and polypropylene/polypropylene carbonate in Greenland ice^33^. Although the number of observations is limited, these initial results suggest that sea ice may function as temporary reservoirs and secondary sources of nanoplastics in the Arctic Ocean, highlighting the need for long-term, vertically resolved observations over wider spatial and temporal scales to better understand their deposition, retention, and release processes and to assess whether the observed patterns are persistent rather than year-specific.

### Potential sources and transportation pathways of nanoplastics in the Arctic Ocean

Nanoplastic concentrations were higher near Svalbard, where local human activities may contribute through river discharge and surface runoff^34–36^. In the open ocean, increased levels in snow and the top layers of ice cores indicated an influence of atmospheric deposition, consistent with long-range aerosol transport reported previously^37,38^. The higher concentrations observed in seawater in 2023 compared with 2022 occurred during a year with more extensive sea ice cover, which may indicate that ice processes facilitate exchanges between the atmosphere, snow/ice, and seawater, while also potentially releasing particles previously retained in ice. In addition, melt ponds may act as transient reservoirs that receive nanoplastics from snowfall and subsequently release them during brine drainage and meltwater percolation^39^. Glacier melt may also release nanoplastics accumulated in glaciers over the past 50 years as a legacy source^40^.

**Fig. 3:**
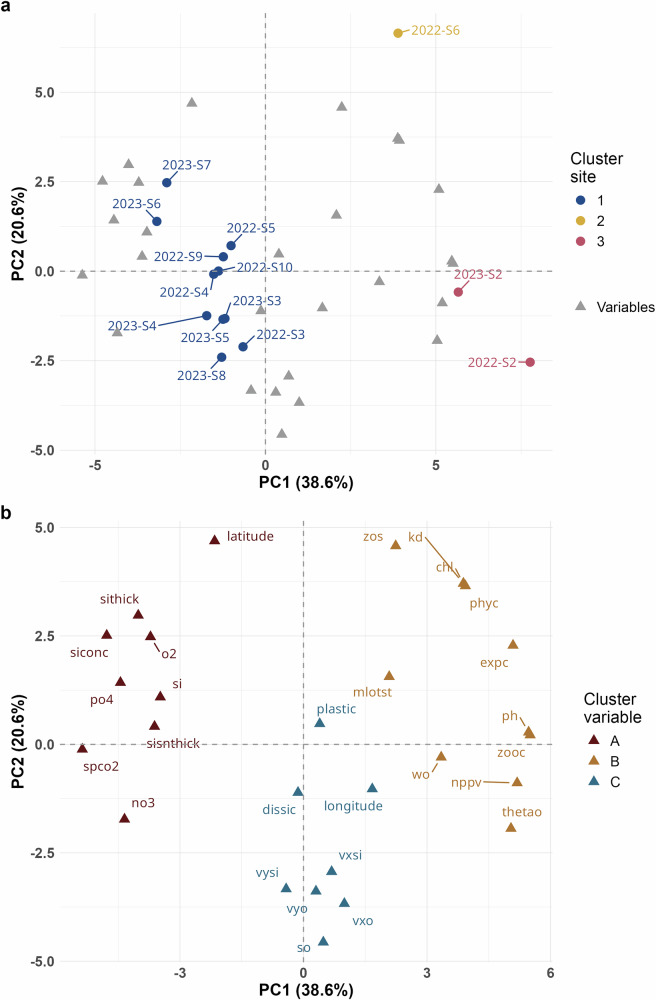
Principal component analysis (PCA) and hierarchical cluster analysis (HCA) of environmental variables and nanoplastic concentrations across sampling sites. **a** Displays the site distribution based on the first two principal components (PC1 and PC2), illustrating the relationship between sampling sites, nanoplastic concentrations, and environmental variables. Sites grouped by the same color indicate clustering based on similar environmental and nanoplastic characteristics according to the HCA results. **b** Shows the interactions among variables, with colors indicating correlated variables identified in the HCA. Detailed information on environmental variables is provided in Table S6, section 4 of the Supplementary Materials.

To investigate the role of oceanic currents as a pathway in the Arctic Ocean, we conducted a statistical analysis of seawater nanoplastics in relation to key oceanographic variables derived from satellite observations and models, covering the same geographic coordinates over 2 years (see “Methods” and Supplementary Materials section 4). Only data of ocean sites were included because of restrictions on protection areas around the archipelago. The principal component analysis (PCA) plot showed the distribution of sampling sites across two principal components (PC1 and PC2), which together account for 59.2% of the total variance (38.6% for PC1 and 20.6% for PC2) (Fig. 3a). PC1 mainly contrasted biological productivity variables (chlorophyll a, phytoplankton carbon, and mixed-layer thickness, with positive loadings) against sea ice related factors (ice concentration, ice thickness, and snow thickness, with negative loadings), indicating a gradient between productive open-water conditions and ice-covered regions. PC2 captured a latitudinal gradient, with positive loadings for latitude, chlorophyll a, and phytoplankton carbon, while negative loadings were associated with seawater and sea ice velocities, reflecting the influence of ocean circulation and ice drift (Fig. 3b).

Notably, sites 2022-S2 and 2023-S2, located at similar latitudes and longitudes north of Svalbard and exposed to higher anthropogenic pollution, provide reasonable supporting evidence for the PCA analysis. Sites located at the North Pole in both years—2022-S6, 2023-S6, and S7—appeared as outliers (Fig. S6c), although 2023-S6 and S7 are positioned closer to the main hierarchical clustering group. These specific sites, located within the Transpolar Drift, have an extra influx of nutrients, organic matter, and nanoplastic pollutants from distal sources, with higher concentrations of certain variables over the year (such as surface snow thickness, sea ice thickness, chlorophyll a, and some nutrients, Table S7), which distinguishes them from other sites. The clustering of several sites on the PCA plot (e.g., 2022-S9, 2022-S10, and 2023 sites) suggested that these sites share similar characteristics across the analyzed variables and are influenced by them to a notable extent.

Nanoplastic concentrations showed only weak loadings on both PC1 (loading = 0.02) and PC2 (loading = 0.03), confirming that their variation is largely independent of the dominant environmental gradients captured by the first two components. This weak correlation suggested that nanoplastic distribution is influenced by additional external drivers not fully represented by the involved variables, resulting in a more sporadic spatial pattern. However, the hierarchical clustering dendrogram revealed that the nanoplastic concentrations correlate weakly with seawater and sea ice velocity (eastward and northward), seawater salinity, longitude, and dissolved inorganic carbon (cluster C). Along PC1, the variation in nanoplastic concentrations was broadly consistent with longitude, seawater current velocity, eastward sea ice velocity, and seawater salinity. In contrast, dissolved inorganic carbon and northward sea ice velocity were inversely related to the variation in nanoplastic concentrations.

These observations point to the involvement of seawater and sea ice circulation in shaping nanoplastic distributions, while also suggesting that additional, unmeasured factors likely contribute to the observed variability. Seawater and sea ice can transport pollutants from distal sources to the central Arctic Ocean and beyond. However, the accelerated retreat of sea ice means that pollutants are accumulating in the central Arctic Ocean or being released in the marginal ice zones^41^. During ice formation, brine rejection increases the salinity of surface seawater, which in turn influences ocean circulation^42^. The results would thus corroborated the findings of our preliminary experimental work^31^, which demonstrate that sea ice plays a dual role as both a storage sink and a release source, functioning as a pivotal medium that exerts a significant influence on the distribution of nanoplastics through its formation and melting processes.

We acknowledge that the limited number of sampling sites and observations introduces a degree of uncertainty, emphasizing the need for long-term and spatially extensive investigations on nanoplastics in the Arctic Ocean. Nanoplastics were widely detected across multiple environmental matrices, with concentrations varying among seawater, snow, melt ponds, and ice cores. These variations reflect the dynamic exchange processes linking the atmosphere, sea ice, and ocean surface. Even though the current dataset represents a limited temporal snapshot, the detection provides compelling evidence that nanoplastic contamination has already reached the Arctic Ocean. The present results therefore represent preliminary, trend-based observations that highlight the urgent need for continued, process-oriented studies integrating physical, chemical, and biological data to better constrain the sources, transport pathways, and environmental fate of nanoplastics in this rapidly changing system.

There is an urgent need to document and understand the distribution and the fate of nanoplastics across the Arctic Ocean, because nanoscale particles can penetrate biological membranes and are much more reactive than their microscale counterparts^43^. This multi-year investigation of nanoplastic distribution in the Arctic Ocean demonstrates (i) differences between globally transported oceanic and local community sources of nanoplastics, especially regarding ice presence relative to the Transpolar Drift and (ii) the increasing contribution of atmospheric deposition as a transport mechanism in remote areas, distant from dense human activity. The range of nanoplastics concentrations is coherent with expected values based on the microplastic concentrations observed in the last 5 years.

Nanoplastics appear to be influenced by parameters that are difficult to measure or model locally, such as seawater and sea ice dynamics, dissolved inorganic matter, and salinity profiles at the ice-sea interface, all of which are crucial to the biogeochemical processes in the Arctic Ocean. The effects of nanoplastic on the biological carbon pump and local biodiversity remain unclear. However, inspired by studies on ultrafine particles (e.g., black carbon, PM0.1, PM1), we believe that specific, yet unrecognized, biological-chemical and physical processes likely occur at the nanoscale, particularly in relation to the distribution of pollutants^44^. It is essential that we develop our ability to measure and model ice-sea interface parameters, which are not included in classical conventional models, to better predict and understand the fate and transportation pathways of nanoplastics in the Arctic Ocean.

Finally, further research is needed to understand their sources, formation, transportation, and transformation across the various compartments of the Arctic Ocean over time. A multitude of environmental factors influence the distribution of nanoplastics. The formation of nanoplastics may occur within milliseconds to months, but aerosol lifetimes range from days to weeks^45^. Meanwhile, particulate matters can travel from the Northern Hemisphere to the Arctic Ocean through thermohaline circulation in 1 year^46,47^. The lifespan of nanoplastics in matrices, their migration pathways, and their inter-matrix transport behaviors all influence the environmental distribution and concentration of nanoplastics. Complex environmental conditions and scenarios drive the continuous partitioning and redistribution of nanoplastics at the sea-ice-air interface, leading to spatial and temporal variations in their environmental concentrations. The findings of our multi-year study, which elucidates the distribution, sources of nanoplastics, and potential transportation pathways, underscore the imperative for the refinement of sampling and monitoring strategies for multi-scaled spatial-and-temporal approaches. These strategies must encompass periods spanning days to years, extend from land to sea, and encompass the ice surface to sediment.

## Methods

### Sample collection and pretreatment

The sampling took place during a round-trip journey from Svalbard archipelago to the geographic North Pole in 2022 and 2023 (Fig. S1). Seawater, ice cores, melt pond water, and snow samples were collected from 24 sites, resulting in a total of 49 samples (Table S1). Onboard pumps were used to collect water samples, which were then transported to the laboratory in 20 L low-density polyethylene (LDPE) containers. These containers were pre-rinsed three times using Milli-Q water before use. Ice cores were collected upwind of the vessel to minimize contamination, using a Mark VI Ice Coring System (1.5 m in length; Kovacs Ice Drilling & Coring Equipment, Roseburg, United States). The top few centimeters of snow or ice were carefully removed during sampling. Each core was cut with a metal blade to separate the upper and lower sections. These sections of the ice cores were placed in metal trays covered with aluminum foil or metallic lids and transferred to the onboard laboratory, where they were allowed to melt at room temperature overnight. Snow and melt pond water samples, when available, were gathered near the ice core locations. Snow samples were collected using a clean metal tray, covered with another tray, and transported to the laboratory aboard the boat. Melt pond water was collected in the same clean 20 L LDPE containers as seawater. The volumes of all samples (seawater, melt-pond water, melted ice cores, and melted snow) were measured on board using a two-liter square bottle (Table S2).

The pretreatment process started with ultrafiltering samples using an Amicon® Stirred Cell (500 mL, Millipore) through 150 kDa (~12.7 nm in hydrodynamic diameter) poly(ether sulfone) filtration membranes (UP150, 1120871, Sterlitech Co.) or a Vivaflow® Tangential Flow Filtration Cassette (50 kDa, ~7.2 nm, 14558321, Sartorius Inc.) to concentrate nanoplastics in the on-board laboratory (Fig. S2). The membrane was rinsed with 5% HCl to remove potential organic interference before use. The samples were concentrated to ~30 mL and then stored in several 7 mL vials (27151, MilliporeSigma™ Supelco™) for later treatment in the laboratory in university. These vials were placed in a freeze-dryer (FreeZone® Bulk Tray Dryer, Labconco Co.) to further concentrate the samples until they became solid, after which the total weight of the solids was recorded. A fraction of solids was transferred to a 20 ml glass vial (Table S2), and subsequently, 10 g of 0.1 M KOH was added to digest the organic matter for 24 h at room temperature. The samples were left to stand for 3 h, after which the supernatant was transferred into a 7 mL glass vial and further concentrated to separate large particles using a centrifuge (4000 rpm, 10 min, 25 °C, Centrifuge 5702, Eppendorf Co.). After centrifugation, the supernatant underwent a dichloromethane extraction, which is considered an efficient method for extracting nanoplastics from environmental samples^48–50^.

### Nanoplastic identification and quantification using pyrolysis-gas chromatography-mass spectrometry

The extracted particles were identified and quantified with pyr-GC-MS (5977B Inert Plus MSD with Intuvo 9000 GC, Agilent Technologies Inc.), following methodologies established in earlier studies^22,51^. Pyrolysis was carried out at 600 °C for 120 s. The GC method was configured with the following parameters: an initial temperature of 50 °C for 2 min, followed by a gradual increase to 180 °C at a rate of 12 °C/min, further elevated to 310 °C at 5 °C/min, and maintained at this temperature for 2 min (Fig. S3). Pyrolysis products were separated on a non-polar gas chromatography column (DB-5MS UI, 60 m × 250 μm × 0.25 μm, Agilent Technologies Inc.). In terms of MS configuration, the ionization voltage was established at 70 eV, and an *m/z* range spanning from 45 to 240 was scrutinized at a scanning frequency of 2000 Hz.

Nanoplastic quantification was developed with internal calibration curves of polystyrene and polypropylene nanoplastics (Fig. S4), which were developed by a series of polystyrene (9003-53-6, Goodfellow Inc.) and polypropylene (Poly-Fil® Poly Pellets®, Fairfield World Company Inc.) standard mixtures, based on the quantifiers at *m/z* = 104 for polystyrene and *m/z* = 70 for polypropylene. Polystyrene beads were dissolved in dichloromethane (DCM, HPLC grade, Thermo Scientific Inc.). Polypropylene beads were dissolved in toluene (HPLC grade, Thermo Scientific Inc.) under reflux. The polypropylene solution was then diluted with DCM to prepare a series of standard mixtures with polystyrene at different concentrations for calibration curve development. To obtain the concentrations of nanoplastics in each sample, the mass of each type of polymer was calculated from the calibration curve and tested volume (Table S2, Equation S1, and Equation S2).

The data processing was conducted on Qualitative Analysis Navigator (Version 10.0, Agilent Technologies Inc.) and MS Quantitative Analysis (Quant-My-Way) (Version B.09.00, Agilent Technologies Inc.). Only the ‘drop baseline’ function was used for data treatment to minimize excessive modification of pyrograms. To identify the pyrolyzate, comparisons of mass spectra with the NIST library spectrum were conducted using NIST MS Search program 2.3 (NIST, Gaithersburg, MD, USA).

### Quality assurance and quality control

Precautionary measures were implemented at each stage, including sample collection, pretreatment, and identification processes. Throughout the entirety of the experimental proceedings, researchers wore nitrile gloves and 100% cotton lab coats to minimize potential fiber or airborne particle contamination. Additionally, each container underwent thorough triple rinsing with Milli-Q water before use.

To assess potential contamination during laboratory analysis, a procedural blank was processed identically to the environmental samples using Milli-Q water in a 20 L LDPE container, covering all analytical steps from ultrafiltration to pyr-GC-MS, to detect any background signals associated with plastic-related compounds. The extracted ion chromatograms showed that no toluene (*m/z* = 91), styrene (*m/z* = 104), 2,4-dimethyl-1-heptene (*m/z* = 55 and 70), or triplet of n-alkadiene/n-alkene/n-alkane (*m/z* = 55, 57, and 83) was found. With the ratio of 91/104 > 1, the ratio of 70/55 ratio < 1, and no triplet presence, it was concluded that no polystyrene, polypropylene or polyethylene was present in the procedural blank. Field blanks were not collected during sampling because of logistical limitations and restricted personnel availability; therefore, the possibility of minor contamination during field collection cannot be entirely excluded.

### Data for environmental variables

The environmental variables were extracted from the publicly available database on the European Union’s Copernicus Marine Service Information. To maintain data consistency, we chose the more comprehensive forecast models for our analysis, which are “Arctic Ocean Biogeochemistry Analysis and Forecast”^52^ and “Arctic Ocean Physics Analysis and Forecast”^53^. The data, provided as daily records with a spatial resolution of 6 km, were processed at each sampling location.

The data were extracted based on specific sampling dates, coordinates, and a depth of 0 m (surface) using R. The R code for the extraction process is publicly available under the MIT license^54^ and archived on Zenodo^55^. After data extraction, variables clearly unrelated to this study, such as the sea water potential temperature at the sea floor (bottomT), sea floor depth below sea level (model_depth), and ocean barotropic streamfunction (stfbaro), were removed. The remaining variables were scaled and then used for further PCA (Table S6). Given the spatial resolution of the remote sensing data (6 km), it was not possible to extract data for the stations that were too close to the shore. Hence, sites that were missing data were excluded from the subsequent PCA (Table S7).

### Principal component analysis, hierarchical cluster analysis, and visualization

A PCA was applied to reduce the dimensionality of the extracted environmental variables to help identify the key components that explain the variance among the observations or sites using R^55^. After standardization and dimensionality reduction of the data, hierarchical clustering analysis was conducted to examine the relationships between sampling sites and environmental variables (see Supplementary Materials section 4, Fig. S6). The resulting dendrogram was used to visualize the similarity between the sampling sites and to identify distinct clusters based on their environmental features.

Other visualizations relied on Python 3 in the freely available Anaconda Navigator (version 2.5.2) using Jupyter Notebook.

## Supplementary information

Supplementary Materials

## Data Availability

The nanoplastic data that support the findings and the presented figures^56^, as well as all the source data extracted from publicly database are available as follow: 10.5281/zenodo.17102673.

## Code availability

Code used to extract oceanographic data, principal component analysis, and hierarchical cluster analysis in this study are available from the Zenodo repository^55^. 10.5281/zenodo.14537222.
