# Supplementary material for: Nanoplastic concentration and potential transport in the Arctic Ocean
Source: NPJ Emerg Contam. 2026 Jan 9;2(1):4. doi: 10.1038/s44454-025-00024-y (PMC12863634; doi:10.1038/s44454-025-00024-y)
Supplement: Supplementary file 1 — Supplementary Materials [file 44454_2025_24_MOESM1_ESM.pdf]

Supplementary Materials for

Nanoplastic Concentration and Potential Transport in the Arctic Ocean

Huiwen Cai[1]*, Charlotte Carrier-Belleau[1], Caroline Guilmette[1], Philippe Massicotte[1], Adèle Luthi-Maire[1], Julien Gigault[1]*

1. Takuvik Laboratory, IRL3376 CNRS-Université Laval, 1045 Avenue de la Médecine, G1V 0A6, Quebéc, QC, Canada

*Corresponding author. Email: huiwen.cai.1@ulaval.ca, julien.gigault@takuvik.ulaval.ca

This PDF file includes:

Sections S1 to S4
Figures S1 to S6
Tables S1 to S7
Equations S1 and S2
Reference 1

Section S1: Sampling

The samples were collected during expedition cruises conducted on *Le Commandant Charcot*, a vessel designed for polar exploration, with scientific missions conducted onboard. The coordinates and names of sampling sites were presented in Figure S1.

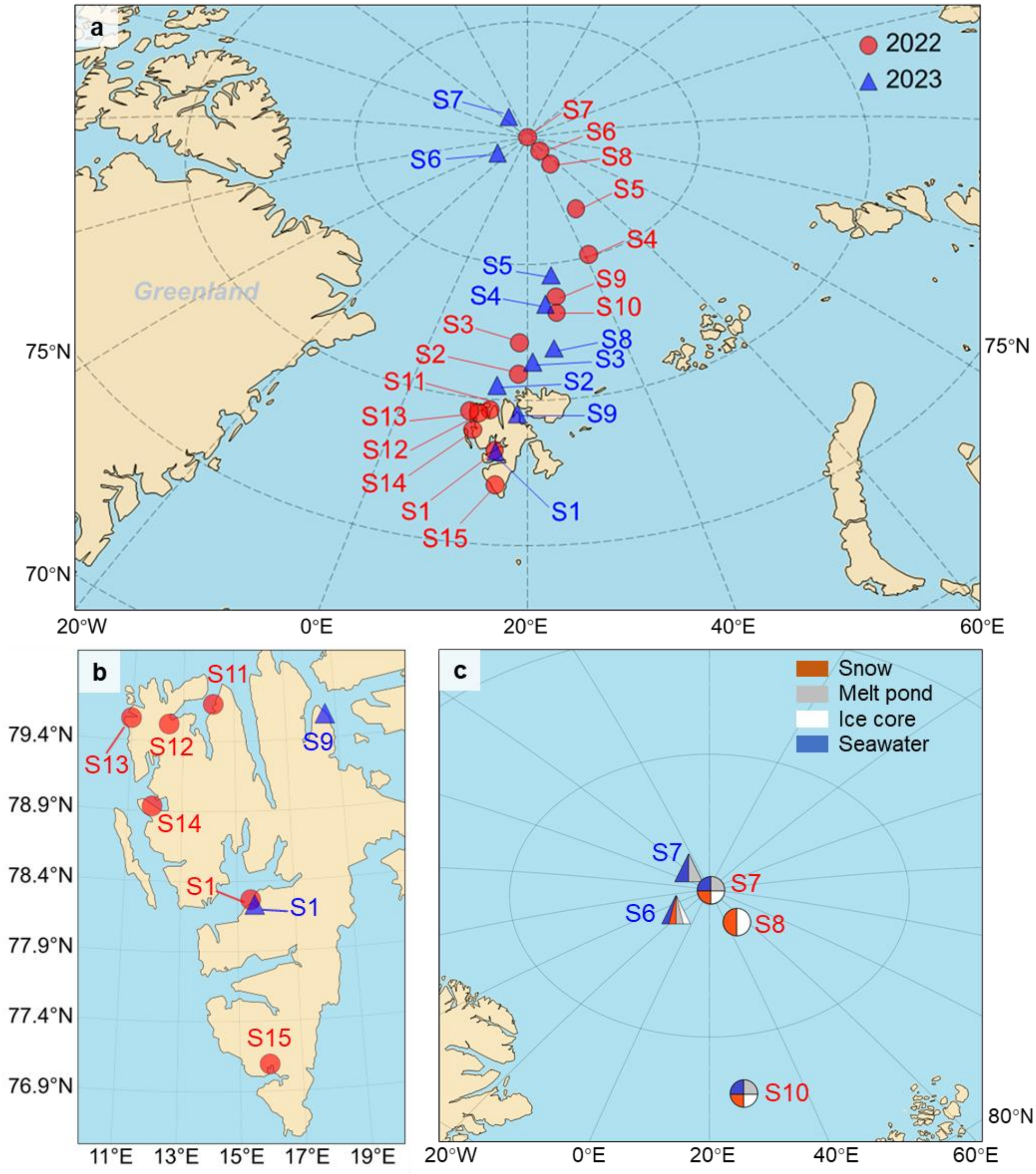

Figure S1: Sampling sites during the summer Charcot expedition in 2022 and 2023. Sampling commenced at the Svalbard port, proceeded north towards the North Pole, and returned south to Burgerbukta. Panel **a**, Top view of the Arctic Ocean showing sampling sites in 2022 (S1-S15) and 2023 (S1-S9). Panel **b**, sampling sites along the coastline of Svalbard archipelago in 2022 (S1, S11-S15) and 2023 (S1 and S9). Panel **c**, sampling sites where different matrices including snow, melt pond, and ice core were collected in 2022 (S6, S7, and S10) and 2023 (S6 and S7). Seawater sampling depth < 3 m.

Table S1. Coordinates of sampling sites and file names.

Site	File name	Latitude (°N)	Longitude (°E)
2022			
S1	CC20220807-1-A	78.2702	15.4603
S2	CC20220808-1-A	80.9569	18.5151
S3	CC20220808-2-V	82.5337	34.0067
S4	CC20220809-1-V	85.0456	40.9784
S5	CC20220810-1-V	86.7759	46.1499
S6	CC20220811-1-V-1L	89.2871	52.4041
	CC20220811-1-V-2L		
	CC20220811-1-V-5L		
	CC20220811-1-V-10L		
	CC20220811-1-V-15L		
	CC20220811-1-V-20L		
S7	CC20220812-1-V	89.9302	46.5465
	CC20220812-1-Top-A		
	CC20220812-1-Bottom-A		
	CC20220812-1-Pond-A		
	CC20220812-1-Snow-A		
S8	CC20220813-1-Top-A	88.6774	51.0378
	CC20220813-1-Bottom-A		
	CC20220813-1-Snow-A		
S9	CC20220815-1-A	83.1400	27.2592
S10	CC20220815-2-V	83.7230	27.7089
	CC20220815-2-Top-A		
	CC20220815-2-Bottom-A		
	CC20220815-2-Pond-A		
	CC20220815-2-Snow-A		
S11	CC20220817-1-V	79.6669	14.1708
S12	CC20220817-2-V	79.5230	12.4441
S13	CC20220818-1V	79.5577	10.9545
S14	CC20220819-1-A	78.9357	11.8833
S15	CC20220820-1-VA	77.0933	15.9511
2023			
S1	CC20230727-A	78.2360	15.6000
S2	CC20230728-1-A	80.5415	14.8683
S3	CC20230728-2-A	81.3958	21.1734
S4	CC20230729-1-A	83.4687	24.7750
S5	CC20230729-2-A	84.5308	27.4434
S6	CC20230731-A	88.9086	-29.9870
	CC20230731-Top-A		
	CC20230731-Bottom-A		
	CC20230731-Pond-A		
	CC20230731-Snow-A		
S7	CC20230801-P1	89.0711	-126.4750

	CC20230801-P2		
	CC20230801-P3		
	CC20230801-P4		
	CC20230801-P5		
	CC20230801-P6		
	CC20230801-Pond		
S8	CC20230805-2-A	81.8530	25.5334
S9	CC20230808-2	79.5711	18.5676

Notes: 1. "Top" refers to the top part of the ice core; "Bottom" is the bottom part of the ice core; and "Pond" refers to samples from the melt pond water on the ice bergs.

Section S2: Pretreatment process of samples.

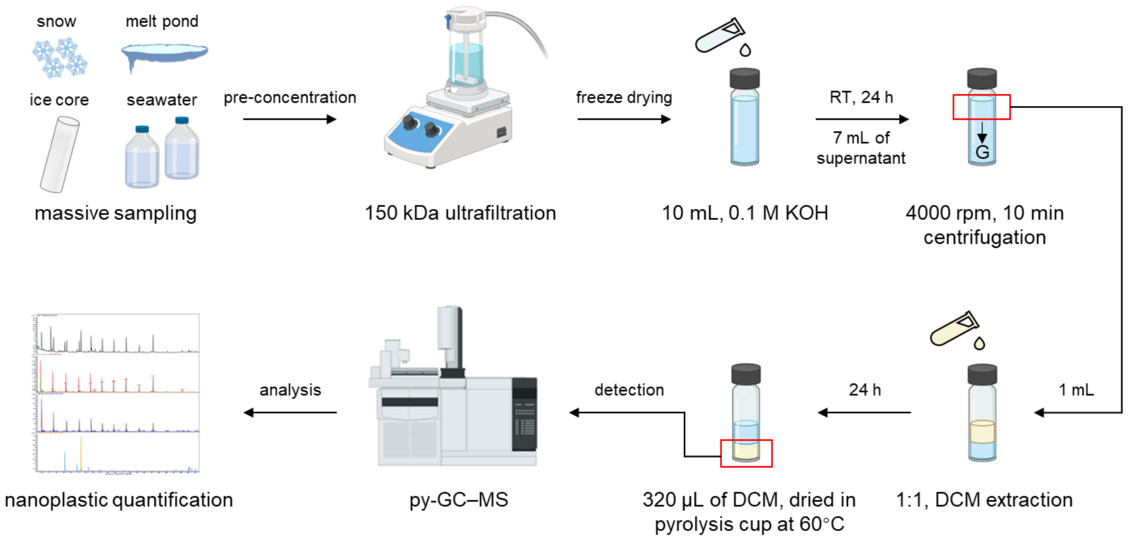

Figure S2: Pretreatment process for nanoplastic detection in environmental samples prior to pyrolysis gas chromatography-mass spectrometry (pyr-GC-MS analysis).

The volume/mass of the samples after each pretreatment step were recorded to measure the aliquot of sample detected and the nanoplastic concentration (Table S2). The calculation is presented in Equation S1:

$$V_{tested} = V_{sample} \times \frac{M_{aliquot}}{M_{pellet}} \times \frac{320\ \mu L}{1000\ \mu L} \qquad (S1)$$

where:

$\boldsymbol{V_{sample}}$ is the volume of seawater and melt pond samples collected from the environment, or the volume of snow and ice cores after melting;

$\boldsymbol{M_{pellet}}$ is the mass of the solid fraction obtained after freeze-drying samples that have been processed through ultrafiltration;

$\boldsymbol{M_{aliquot}}$ is the mass of sub-samples taken from the pellets; and

$\frac{320\ \mu L}{1000\ \mu L}$ is the coefficient of the DCM fraction injected in pyr-GC-MS out of 1000 µL DCM for extraction.

Table S2: Volume of samples when sampling (V_{sample}), mass of pellets after freeze-drying (M_{pellet}), mass of aliquots taken after centrifugation ($M_{aliquot}$), and volume of samples tested (V_{tested}) with pyr-GC-MS.

Site	File name	V_{sample} (L)	M_{pellet} (g)	$M_{aliquot}$ (g)	V_{tested} (L)
2022					
S1	CC20220807-1-A	21.0	1.232	0.197	1.075
S2	CC20220808-1-A	20.0	1.730	0.200	0.740
S3	CC20220808-2-V	20.0	0.340	0.207	3.902
S4	CC20220809-1-V	20.0	1.348	0.194	0.923
S5	CC20220810-1-V	14.8	0.400	0.180	2.134
S6	CC20220811-1-V-20L	20.0	1.232	0.197	4.664
S7	CC20220812-1-V	20.0	0.289	0.211	1.270
	CC20220812-1-Top-A	6.2	-	-	6.200
	CC20220812-1-Bottom-A	5.5	-	-	5.500
	CC20220812-1-Pond-A	19.6	-	-	19.600
	CC20220812-1-Neige-A	13.8	-	-	13.800
S8	CC20220813-1-Top-A	4.0	-	-	4.000
	CC20220813-1-Bottom-A	3.6	-	-	3.600
	CC20220813-1-Neige-A	5.3	-	-	5.300
S9	CC20220815-1-A	18.0	1.000	0.212	1.222
S10	CC20220815-2-V	20.0	0.233	0.204	5.606
	CC20220815-2-Top-A	8.2	-	-	8.200
	CC20220815-2-Bottom-A	8.9	-	-	8.900
	CC20220815-2-Pond-A	14.5	0.988	0.211	0.989
	CC20220815-2-Neige-A	4.7	-	-	4.700
S11	CC20220817-1-V	18.0	0.200	0.103	2.961
S12	CC20220817-2-V	12.0	0.699	0.212	1.162
S13	CC20220818-1V	9.0	1.542	0.210	0.392
S14	CC20220819-1-A	8.0	0.725	0.183	0.645
S15	CC20220820-1-VA	18.0	0.798	0.209	1.506
2023					
S1	CC20230727-A	20.0	-	-	2.133
S2	CC20230728-1-A	20.0	-	-	1.600
S3	CC20230728-2-A	21.5	-	-	1.720
S4	CC20230729-1-A	20.0	-	-	1.600
S5	CC20230729-2-A	20.0	-	-	1.600
S6	CC20230731-A	19.0	-	-	1.520
	CC20230731-Top-A	7.3	-	-	0.779
	CC20230731-Bottom-A	10.3	-	-	0.412
	CC20230731-Pond-A	18.3	-	-	2.928
	CC20230731-Snow-A	17.4	-	-	2.776

S7	CC20230801-P1	22.0	-	-	3.520
	CC20230801-P2	24.2	-	-	3.872
	CC20230801-P3	25.0	-	-	4.000
	CC20230801-P4	21.2	-	-	3.392
	CC20230801-P5	23.6	-	-	3.776
	CC20230801-P6	22.0	-	-	3.520
	CC20230801-Pond	49.50	-	-	6.336
S8	CC20230805-2-A	16.0	-	-	1.280
S9	CC20230808-2	4.0	-	-	0.640

Notes: 1. Aliquot is the sub-samples taken from freeze-dried pellets.

2. Top refers to the top part of the ice core; "Bottom" is the bottom part of the ice core; and "Pond" means water samples taken from the melt pond on the ice bergs; letter A is water ultrafiltered by Amicon; letter V is water ultrafiltered by Vivaflow.

3. "-" means that the weight of pellets or aliquots are too low to measure. All the material in vials were rinsed off with 0.1 M of KOH for pretreatment and analysis.

Section S3: Identification and quantification of nanoplastic

Identification

The extracted particles were identified and quantified with pyr-GC-MS.

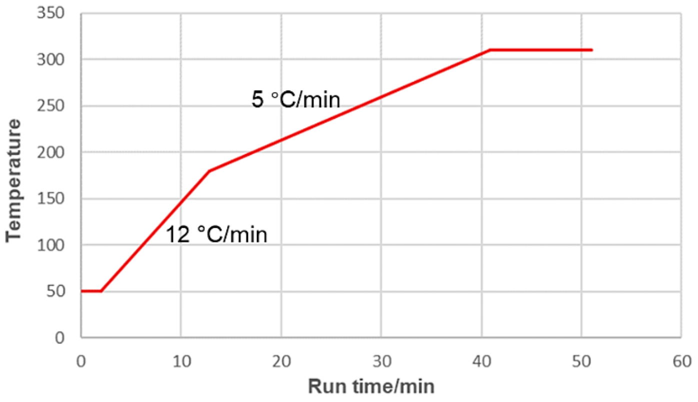

Figure S3: The temperature programming chart for nanoplastic identification using pyr-GC-MS

In addition to the toluene/styrene ratio for polystyrene (PS) identification described in the main text, an *m/z* = 70 to *m/z* = 55 ratio was used for polypropylene (PP). Based on the PP pyrogram from the NIST MS library (Version 2.3, Gaithersburg, MD, USA) and laboratory analysis of PS and PP standards, the threshold for this ratio was set at > 1, accounting for the limit of quantification for PP on MS (15 ng) and an almost negligible response value (Deut_0,2_2_PP_1_25_PS_1_5_R3_20240506) (grey cells in Table S3).

Table S3: Target response (TR) and retention time (RT) of *m/z* = 70 and *m/z* = 55 extracted from PP and PS standards, along with their corresponding ratios.

File name	*m/z* = 70		*m/z* = 55		70/55
	RT	TR	RT	TR	

Deut_0,2_2_PP_1_5_PS_1_1_R2_20240506	6.988	581449	6.999	687736	0.845454
Deut_0,2_2_PP_1_5_PS_1_1_R3_20240506	6.989	356149	6.991	416282	0.855547
Deut_0,2_2_PP_1_5_PS_1_1_R1_20240506	6.996	340130	6.999	318473	1.068003
Deut_0,2_2_PP_1_25_PS_1_5_R3_20240506	6.991	1050215	6.992	758685	1.384257
Deut_0,2_2_PP_1_50_PS_1_10_R3_20240507	6.990	1757965	6.990	1218677	1.442519
Deut_0,2_2_PP_1_75_PS_1_15_R2_20240508	6.987	3663645	6.987	2536004	1.444653
Deut_0,2_2_PP_1_25_PS_1_5_R1_20240506	6.989	927263	6.993	629879	1.472129
Deut_0,2_2_PP_1_75_PS_1_15_R5_20240508	6.983	3001335	6.985	1896982	1.582163
Deut_0,2_2_PP_1_75_PS_1_15_R3_20240508	6.987	3116010	6.987	1840133	1.693361
Deut_0,2_2_PP_1_50_PS_1_10_R1_20240507	6.988	1946370	6.989	1109796	1.753809
Deut_0,2_2_PP_10_100_PS_1_20_R3_20240507	6.989	3621533	6.988	2037473	1.777463
Deut_0,2_2_PP_10_100_PS_1_20_R2_20240507	6.989	4025229	6.989	2211994	1.819729
Deut_0,2_2_PP_1_50_PS_1_10_R2_20240507	6.992	1863696	6.991	1021512	1.824448
Deut_0,2_2_PP_10_150_PS_1_30_R3_20240508	6.99	7568218	6.991	4141251	1.82752
Deut_0,2_2_PP_10_150_PS_1_30_R2_20240508	6.971	7444674	6.971	3968437	1.875971
Deut_0,2_2_PP_10_150_PS_1_30_R1_20240508	6.990	6069027	6.990	3223352	1.882831
Deut_0,2_2_PP_1_25_PS_1_5_R2_20240506	6.991	1183369	6.990	624115	1.896075
Deut_0,2_2_PP_10_200_PS_1_40_R2_20240507	6.991	10281806	6.991	5403516	1.902799
Deut_0,2_2_PP_10_100_PS_1_20_R1_20240507	6.993	4001615	6.991	2085417	1.918856
Deut_0,2_2_PP_10_200_PS_1_40_R1_20240507	6.988	9450654	6.988	4890219	1.932563
Deut_0,2_2_PP_10_350_PS_1_60_R2_20240507	6.989	19890806	6.989	10247917	1.940961
Deut_0,2_2_PP_10_200_PS_1_40_R3_20240507	6.961	11172783	6.962	5750553	1.942906
Deut_0,2_2_PP_10_275_PS_1_50_R1_20240508	6.990	13776693	6.989	7077373	1.946583
Deut_0,2_2_PP_10_275_PS_1_50_R3_20240508	6.990	14597980	6.990	7339822	1.988874
Deut_0,2_2_PP_10_350_PS_1_60_R1_20240507	6.990	19193400	6.991	9592126	2.000954
Deut_0,2_2_PP_10_350_PS_1_60_R3_20240507	6.990	18557683	6.991	9251156	2.005985
Deut_0,2_2_PP_10_275_PS_1_50_R2_20240508	6.991	16536603	6.991	8018693	2.062257
Deut_0,2_2_PP_1_25_PS_1_5_R3_20240506	6.987	5893	7.007	2294	2.568875

Quantification

The quantification work is unpublished; however, the developed calibration curves incorporate PS and PP plastic standards at the same time, making them well-suited for quantifying nanoplastics in this study.

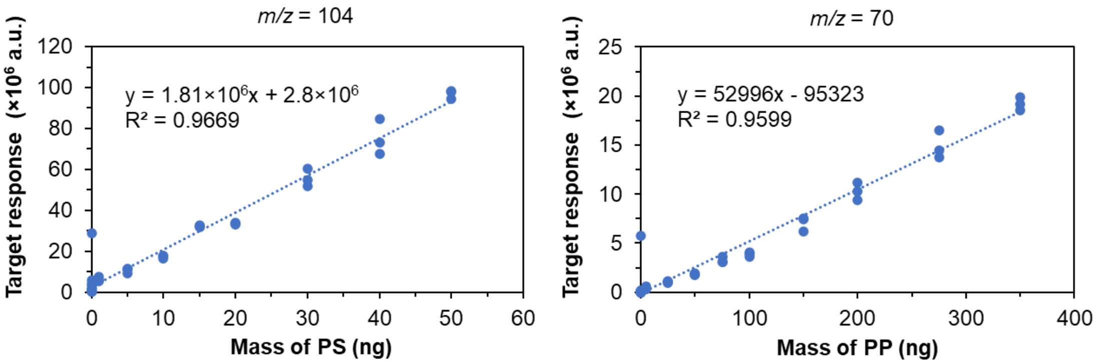

Figure S4: Calibration curves for nanoplastics based on m/z = 104 for PS and m/z = 70 for PP.

Since the calibration method for quantification differs from that used for sample detection, a data conversion (Equation S2) was applied to calculate the target response (TR) in each sample:

$$TR_{calibrated(m/z=i)} = TR_{sample(m/z=i)} \times \frac{\overline{TR}_{std.D(q,m/z=104)}}{\overline{TR}_{std.D(s,m/z=104)}} \times \frac{TR_{std.quantiM(m/z=104)}}{TR_{std.sampleM(m/z=104)}} \qquad \text{(S2)}$$

where:

$TR_{calibrated(m/z=i)}$ is the TR at *m/z* = *i* after calibration, specifically *m/z* = 104 or *m/z* = 70 which are quantifiers for PS or PP;

$TR_{sample(m/z=i)}$ is the TR at *m/z* = *i* of samples using sample detection method; $\overline{TR}_{std.D(q,m/z=104)}$ is the average TR at *m/z* = 104 of morning PS standards during the quantification days;

$\overline{TR}_{std.D(t,m/z=104)}$ is the average TR at *m/z* = 104 of morning PS standards during the sample detection days;

$TR_{std.quantiM(m/z=104)}$ is the TR at *m/z* = 104 of PS standards in the sample using quantification method; and

$TR_{std.sampleM(m/z=104)}$ is the TR at *m/z* = 104 of PS standards in the sample using sample detection method. The first ratio in the equation represents a conversion over days using a 20 ng PS standard to monitor machine status, while the second ratio provides a conversion between methods.

Certain PS and PP masses exceeded the calibration range, but the overall conclusions are unaffected because they depend on relative trends rather than absolute values. Due to the current lack of reliable quantifiers for PE nanoplastics, only an "identified" label is included in the table to indicate the presence of PE in the samples. All the nanoplastic identification and quantification data processing are available on Zenodo[1].

Table S4: PS and PP nanoplastic concentration, and PE detected at each sampling site.

Site	File name	PS (ng L^{-1})	PP (ng L^{-1})	PE (ng L^{-1})	Sum (ng L^{-1})
2022					
S1	CC20220807-1-A	51.4	245.7	identified	297.1
S2	CC20220808-1-A	54.0	133.2	-	187.2
S3	CC20220808-2-V	0.0	103.2	-	103.2
S4	CC20220809-1-V	0.0	166.5	-	166.5
S5	CC20220810-1-V	15.7	84.9	-	100.6
S6	CC20220811-1-V-20L	94.0	161.4	identified	255.4
S7	CC20220812-1-V	22.4	98.2	-	120.6
	CC20220812-1-Top-A	12.7	61.9	-	74.6
	CC20220812-1-Bottom-A	0.0	37.8	-	37.8
	CC20220812-1-Pond-A	28.3	144.3	identified	172.6
	CC20220812-1-Snow-A	332.9	99.4	identified	432.3
S8	CC20220813-1-Top-A	34.1	87.9	-	122.0
	CC20220813-1-Bottom-A	0.0	0.0	-	0.0

	CC20220813-1-Snow-A	0.0	0.0	-	0.0
S9	CC20220815-1-A	30.1	190.9	-	221.0
S10	CC20220815-2-V	0.0	14.2	-	14.2
	CC20220815-2-Top-A	0.0	96.9	-	96.9
	CC20220815-2-Bottom-A	44.4	90.1	identified	134.5
	CC20220815-2-Pond-A	0.0	131.22	identified	131.2
	CC20220815-2-Snow-A	0.0	0.0	-	0.00
S11	CC20220817-1-V	0.0	18.5	-	18.5
S12	CC20220817-2-V	0.0	79.1	-	79.1
S13	CC20220818-1V	121.7	179.8	-	301.6
S14	CC20220819-1-A	0.0	179.8	-	179.8
S15	CC20220820-1-VA	0.0	55.4	-	55.4
2023					
S1	CC20230727-A	33.0	92.2	identified	125.3
S2	CC20230728-1-A	45.4	107.3	-	152.7
S3	CC20230728-2-A	34.4	103.0	identified	137.4
S4	CC20230729-1-A	0.0	327.8	identified	327.8
S5	CC20230729-2-A	44.9	119.7	-	164.6
S6	CC20230731-A	94.2	113.4	-	207.6
	CC20230731-Top-A	314.3	672.0	identified	986.3
	CC20230731-Bottom-A	0.0	0.00	identified	0.0
	CC20230731-Pond-A	314.3	52.5	-	59.8
	CC20230731-Snow-A	87.0	158.1	identified	245.1
S7	CC20230801-P1	74.6	44.1		-
	CC20230801-P2	348.3	41.9	identified	-
	CC20230801-P3	5.2	96.6	-	-
	CC20230801-P4	17.2	159.6	-	-
	CC20230801-P5	0.0	52.0	-	-
	CC20230801-P6	0.0	0.0	identified	-
	CC20230801-$P_{Average}$	74.2	65.7	-	139.9
	CC20230801-Pond	7.8	21.8	-	29.6
S8	CC20230805-2-A	204.1	78.1	identified	282.2
S9	CC20230808-2	492.5	265.6	identified	758.1

Table S5: Average nanoplastic concentration of ocean sites and coastline sites for 2022 and 2023.

Site	Average nanoplastic concentration (ng L^{-1})
Coastline sites	
2022-S1, S2 and S11–S15	159.8
2023-S1, S2 and S9	345.4
Ocean sites	
2022-S3–S7, S9 and S10	140.2
2023-S3–S8	209.9

Quality assurance and quality control

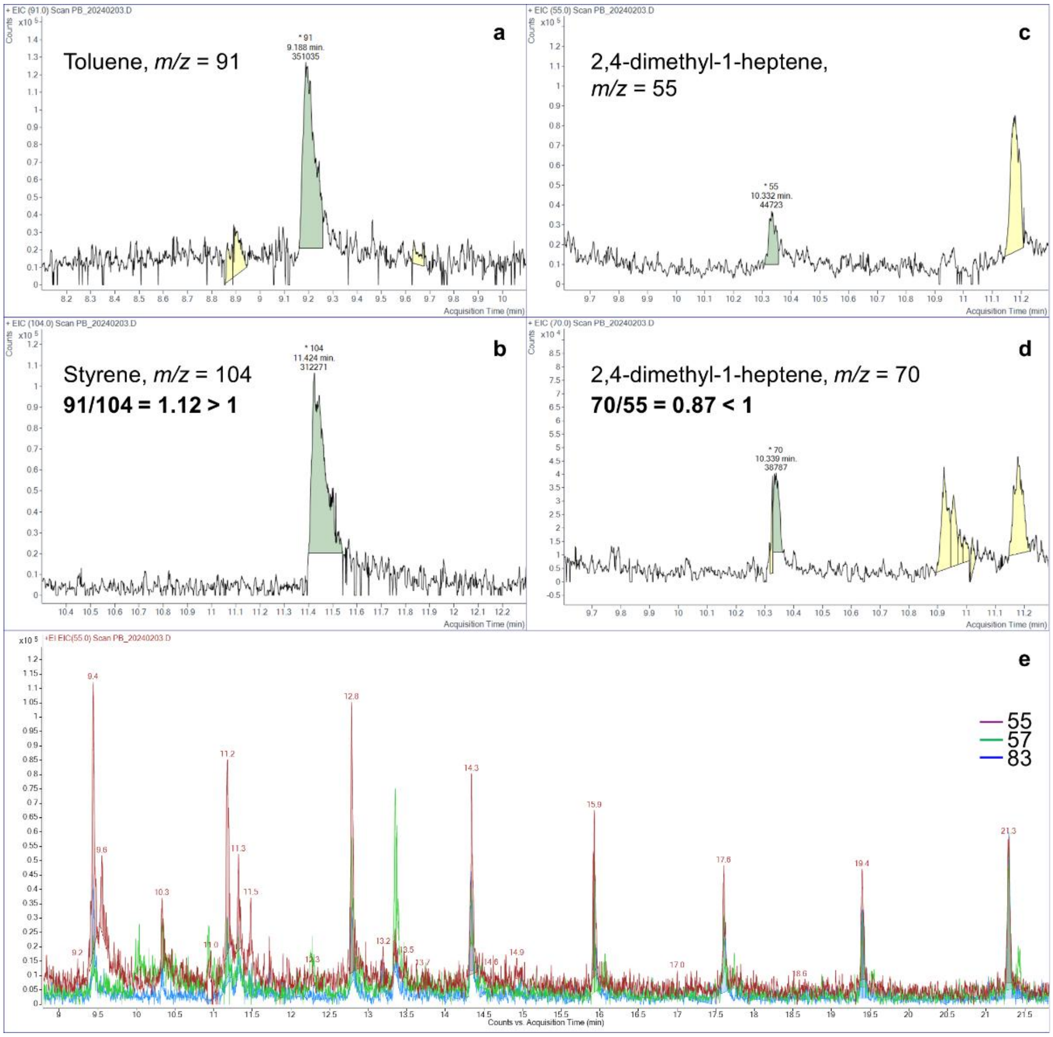

Figure S5: Integrated peaks in the extracted ion chromatograms for PS: (a) *m/z* = 91, (b) *m/z* = 104, for PP: (c) *m/z* = 55, (d) *m/z* = 70, and for PE: (e) *m/z* = 55, 57, and 83 in the procedural blank sample.

Section S4: Data extraction from online database

Environmental variables data

We used the European Union's Copernicus Marine Service Information for the environmental variables to analyse oceanographic conditions in the Arctic.

Table S6: Environmental variables and abbreviations.

Abbreviation	Variable	Unit
plastic	nanoplastic	ng L^{-1}
mlotst	ocean mixed layer thickness defined by sigma theta	m
siconc	sea ice area fraction	-

sisnthick	surface snow thickness	m
sithick	sea ice thickness	m
so	sea water salinity	10^{-3}
thetao	sea water potential temperature	°C
vxo	eastward sea water velocity	m s^{-1}
vxsi	eastward sea ice velocity	m s^{-1}
vyo	northward sea water velocity	m s^{-1}
vysi	northward sea ice velocity	m s^{-1}
wo	upward sea water velocity	m s^{-1}
zos	sea surface height above geoid	m
chl	mass concentration of chlorophyll a in sea water	mg m^{-3}
dissic	mole concentration of dissolved inorganic carbon in sea water	mole m^{-3}
expc	sinking mole flux of particulate organic matter expressed as carbon in sea water	mol m^{-2} d^{-1}
kd	volume attenuation coefficient of downwelling radiative flux in sea water	m^{-1}
NO_3	mole concentration of nitrate in sea water	mmol m^{-3}
nppv	net primary production of biomass expressed as carbon per unit volume in sea water	mg m^{-3} day^{-1}
O_2	mole concentration of dissolved molecular oxygen in sea water	mmol m^{-3}
pH	sea water pH reported on total scale	-
phyc	mole concentration of phytoplankton expressed as carbon in sea water	mmol m^{-3}
PO_4	mole concentration of phosphate in sea water	mmol m^{-3}
Si	mole concentration of silicate in sea water	mmol m^{-3}
$spCO_2$	surface partial pressure of carbon dioxide in sea water	Pa
zooc	mole concentration of zooplankton expressed as carbon in sea water	mmol m^{-3}

Valid environmental variables at each sampling site are presented in Table S7.

Table S7: Environmental variables and sampling sites with valid data.

Variable	2022-S2	2022-S3	2022-S4	2022-S5	2022-S6	2022-S9	2022-S10
longitude	18.5151	34.0067	40.9784	46.1499	52.4041	27.2592	27.7089
latitude	80.9569	82.5337	85.0456	86.7759	89.2871	83.1400	83.7230
plastic	182.07	103.21	166.52	99.05	246.45	14.17	218.07
mlotst	8.747775	3.499099	3.922183	3.745006	5.612463	4.913730	6.606895
siconc	0.000000	0.409862	0.877832	0.785294	0.864401	0.928252	0.925186
sisnthick	0.000000	0.018323	0.023697	0.019200	0.019881	0.016549	0.012942
sithick	0.000000	0.416927	1.656121	1.770660	2.071389	1.214873	1.545033
so	33.737598	31.823698	32.673977	31.987774	29.909277	31.579092	32.449539
thetao	5.161159	-0.737955	-1.716318	-1.683061	-1.597464	-1.667744	-1.720536
vxo	0.029909	0.015874	-0.064453	-0.072277	-0.052171	-0.062674	-0.043636
vxsi	0.000000	-0.013509	-0.033300	-0.049486	-0.024769	-0.016087	0.001961
vyo	0.042663	0.045077	-0.026580	-0.005710	-0.046260	-0.049920	-0.050613
vysi	0.000000	0.021335	-0.103274	-0.066000	-0.126499	-0.138720	-0.140629

wo	0.000000	0.000000	0.000000	0.000000	0.000000	0.000000	0.000000
zos	-0.604708	-0.622137	-0.646470	-0.581662	-0.487484	-0.633561	-0.639279
chl	0.676832	0.002550	0.001459	0.007039	2.099747	0.001014	0.000755
dissic	2.216631	2.267995	2.261506	2.283224	2.248287	2.267673	2.265161
expc	3.855079	0.003394	0.001328	0.009189	4.498295	0.001728	0.001287
kd	0.068073	0.041102	0.041058	0.041282	0.124990	0.041041	0.041030
NO_3	0.073260	5.864423	5.686665	4.120975	1.924375	4.908603	4.902117
nppv	12.906280	0.016938	0.004689	0.031355	3.379449	0.005851	0.003166
O_2	328.76287	378.38836	378.42880	379.70962	383.72586	378.90869	378.55862
pH	8.100317	8.009016	8.001265	8.010317	8.080505	7.997209	7.995739
phyc	3.586750	0.012042	0.005637	0.028041	10.594945	0.004044	0.002697
PO_4	0.121294	0.472866	0.454271	0.394391	0.309326	0.408376	0.405862
Si	1.382619	4.284532	4.671668	3.087486	1.008953	5.543825	5.118309
$spCO_2$	32.538574	39.778580	40.358566	39.989143	34.595875	40.889751	40.946144
zooc	0.959376	0.000166	0.000166	0.000166	0.530429	0.000167	0.000167

**Variable**	**2023-S2**	**2023-S3**	**2023-S4**	**2023-S5**	**2023-S6**	**2023-S7**	**2023-S8**
longitude	14.8683	21.1739	24.7750	27.4434	-29.9875	-126.4754	25.5334
latitude	80.5415	81.3958	83.4686	84.5308	88.9086	89.0711	81.8530
plastic	148.39	134.10	327.79	160.39	198.76	132.88	262.97
mlotst	6.558871	3.556241	4.012604	4.169811	6.577624	9.436947	3.638546
siconc	0.110288	0.840057	0.797009	0.776605	0.979996	0.970503	0.837322
sisnthick	0.020158	0.051379	0.052711	0.052755	0.056031	0.056816	0.029764
sithick	0.158638	1.613695	1.888271	1.892394	2.025150	2.001753	1.571408
so	32.266613	31.955210	32.926285	33.174221	32.100506	31.714607	32.161675
thetao	2.659991	-1.577097	-1.672807	-1.655639	-1.728568	-1.708151	-1.633485
vxo	0.008039	0.035501	0.015035	0.031411	-0.011270	-0.027429	0.051917
vxsi	0.019437	0.050484	0.050090	0.039425	-0.068130	-0.020509	0.028023
vyo	-0.025759	0.008017	-0.012397	0.000398	0.019586	-0.010398	0.044198
vysi	-0.040085	0.001617	0.010435	0.014364	0.053223	-0.085830	0.106186
wo	0.000000	0.000000	0.000000	0.000000	0.000000	0.000000	0.000000
zos	-0.592807	-0.608660	-0.609885	-0.609389	-0.599364	-0.587427	-0.658100
chl	0.656508	0.132185	0.031406	0.019862	0.013705	0.054831	0.082017
dissic	2.229937	2.253025	2.244586	2.249552	2.178324	2.176172	2.246318
expc	2.143757	0.095100	0.051388	0.038380	0.077325	0.223014	0.073082
kd	0.067260	0.046287	0.042256	0.041794	0.041548	0.043193	0.044281
NO_3	1.320285	5.054921	6.588175	3.315251	3.269330	3.236317	6.475452
nppv	5.398982	1.966755	0.355779	0.272929	0.197869	0.755013	0.961598
O_2	381.82800	378.66595	377.42187	377.13427	384.99447	385.18719	378.91555
ph	8.142196	8.021056	7.995767	7.999245	7.981409	7.984203	8.010208
phyc	3.388409	0.803671	0.190015	0.119956	0.082419	0.334991	0.499563
PO_4	0.137521	0.367031	0.523504	0.342834	0.892712	0.888465	0.498420
Si	1.978873	5.772206	7.170250	5.810882	21.313910	21.366899	7.108721
$spCO_2$	28.708868	38.483250	40.527996	40.288406	41.423859	41.112415	39.353668
zooc	1.091849	0.001696	0.001664	0.001664	0.001665	0.001664	0.001663

Principal component analysis and hierarchical cluster analysis

To facilitate intuitive visualisation of the principal component analysis results, the first two principal components were retained (Figure S6a). Subsequently, a hierarchical cluster analysis was conducted on the sites (Figure S6c) and variables (Figure S6d). The optimal

number of clusters was determined through the evaluation of clustering performance using the silhouette score method (Figure S6b).

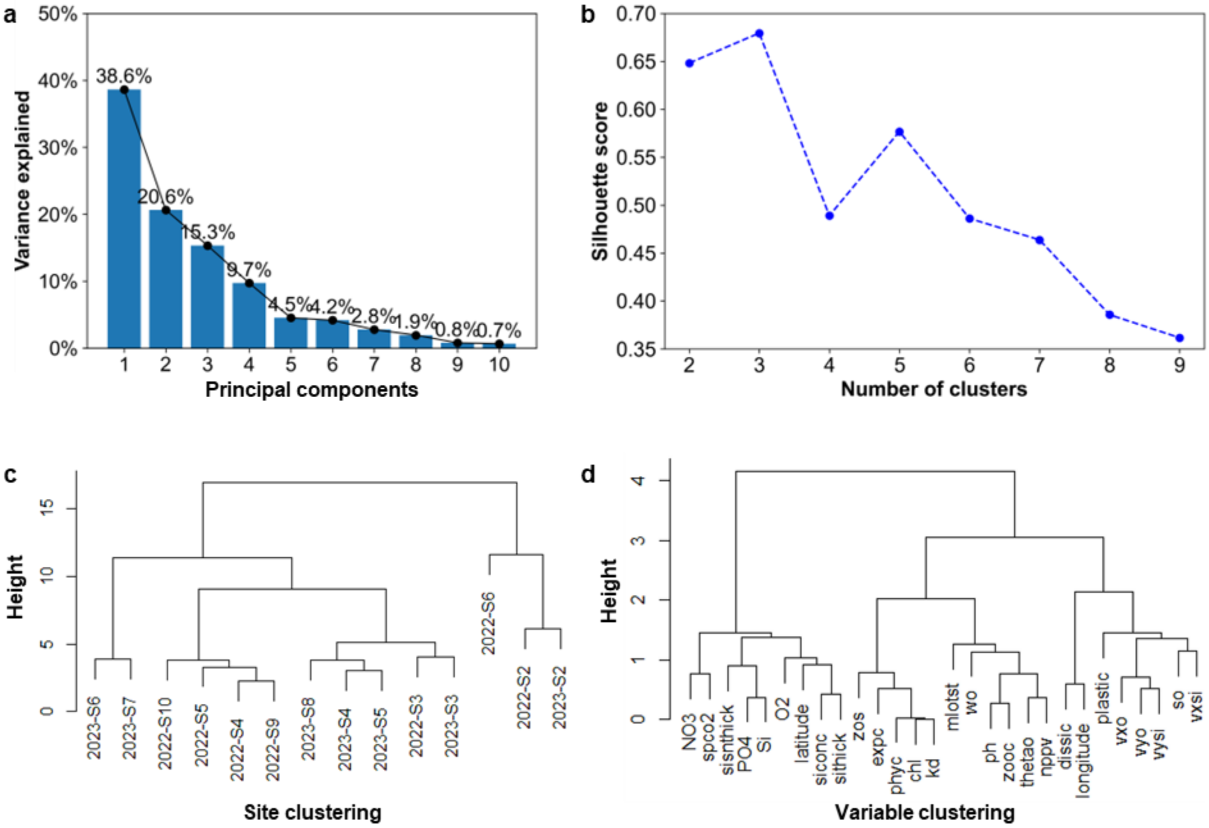

Figure S6: Principal component analysis and hierarchical cluster analysis for sites, nanoplastic concentrations and environmental variables. Panel **a**, variances explained along with number of principal components after dimensionality reduction. Panel **b**, silhouette scores for each number of clusters. The overall score of the clustering scheme was evaluated by calculating the mean silhouette coefficient across all samples. Panel **c**, the hierarchical clustering for sites after dimensionality reduction. Panel **d**, the hierarchical clustering for nanoplastic concentration and environmental variables after dimensionality reduction.

References

1. Cai, H. *et al.* Nanoplastics in the Arctic Ocean-Dataset. Zenodo https://doi.org/10.5281/ZENODO.17102673 (2025).
